# Efficacy and Safety of Intranasal Dexmedetomidine vs. Oral Chloral Hydrate for Sedation in Children Undergoing Computed Tomography/Magnetic Resonance Imaging: A Meta-Analysis

**DOI:** 10.3389/fped.2022.872900

**Published:** 2022-03-31

**Authors:** Xiaoqian Lyu, Yujuan Tao, Xiujing Dang

**Affiliations:** ^1^Department of Anaesthesiology, Sanya Women and Children’s Hospital Managed by Shanghai Children’s Medical Center, Sanya, China; ^2^Department of Anaesthesiology, Qilu Children’s Hospital of Shandong University, Jinan, China

**Keywords:** dexmedetomidine (DEX), chloral hydrate, efficacy, meta-analysis, review

## Abstract

**Objective:**

This meta-analysis aims to evaluate the sedative efficacy and safety of intranasal administration of dexmedetomidine (DEX) compared with oral chloral hydrate for Computed tomography (CT) or Magnetic Resonance Imaging (MRI) examination in Children.

**Methods:**

Cochrane Library, PubMed, Embase, Web of Science, China National Knowledge Infrastructure (CNKI), and China WanFang Databases were searched to collect randomized controlled trials (RCTs) investigating intranasal DEX (test group) vs. oral chloral hydrate (control group) in pediatric CT/MRI examinations up to December 30, 2021. The data were analyzed using Stata 15.0 software.

**Results:**

Seven RCTs with 1,846 children were identified. The meta-analysis results showed that the success rate of sedation (RR = 1.14, 95% CI: 1.03–1.26, *P* = 0.011), sedation onset time [weighted mean difference (WMD) = –0.87, 95% CI: –1.42 to –0.31, *P* = 0.002], sedation duration (WMD = –9.05, 95% CI:-14.69 to –3.42, *P* = 0.002), time to awakening (WMD = –9.75, 95% CI:-17.57 to –1.94, *P* = 0.014), and incidence of nausea and vomiting [relative risk (RR) = 0.09, 95% CI:0.04–0.23, *P* < 0.001) of the test group were significantly better than those of the control group. However, no significant differences were identified in incidence of hypotension (RR = 1.18, 95% CI: 0.51–2.74) and bradycardia (RR = 1.17, 95% CI: 0.13–22.11) between the two groups.

**Conclusion:**

Intranasal administration of DEX is superior to oral chloral hydrate for sedation during pediatric CT/MRI examinations and has a better safety profile.

## Introduction

Children, a particular medical group, have poor tolerance to unfamiliar environments or things and poor emotional control because of their young age, incomplete physical and mental development. Therefore, pediatric patients are prone to uncooperative behaviors such as crying and moving during Computed tomography (CT) or Magnetic Resonance Imaging (MRI) and other examinations associated with various equipment noises as well as cold, dim and claustrophobic environments. Such adverse emotions and uncooperation will significantly affect the implementation and effect of imaging examinations (1). Anxiety and fear can lead to increasing catecholamine levels in the body, causing tachycardia, increased airway secretions, shortness of breath; furthermore, the difficulty for children to separate from their parents and complete imaging examinations is risen (2). Given the above reasons, it is necessary to perform moderate to deep sedation or anesthesia in children, especially infants and preschool children, to alleviate the anxiety as much as possible during imaging examinations, maintain absolute immobilization, reduce the production of motion artifacts, and ultimately improve the quality of CT or MRI imaging (3, 4).

Chloral hydrate, a sedative/hypnotic drug, has a significant sedative effect and is commonly used for children (5, 6). However, this drug still has limitations in pediatric sedation. Chloral hydrate has a pungent, spicy odor and a slightly bitter taste, so some children show resistance to oral chloral hydrate; this oral drug also irritates the gastrointestinal tract and can lead to nausea, vomiting, and other discomforts in children (7). Additionally, chloral hydrate, like most sedative drugs, has irritability after awakening. With the accumulated clinical experience of its use, severe adverse reactions such as laryngospasm and respiratory depression have been reported after the use of chloral hydrate in children (8). Therefore, it is particularly vital to seek a sedative drug with higher safety and efficacy to assist children in completing auxiliary examinations such as MRI.

Dexmedetomidine (DEX) hydrochloride can exert a sedative effect by activating α2 adrenoceptors, and its speed of onset is faster than that of traditional sedative drugs. Also, it has a relatively short period of drug half-life in blood and drug accumulation in the body. Moreover, DEX hydrochloride can also play a certain anxiolytic effect by inhibiting sympathetic nerve activity, with fewer side effects (9). As a result, this sedative drug has been paid more and more attention clinically. DEX hydrochloride induces average physiological sleep onset to achieve sedation for children, so it is theoretically safe and has good feasibility in pediatric examinations. However, considering that DEX has some anti-sympathetic effect, the possibility of causing bradycardia and hypotension theoretically cannot be ignored when used as a sedative for sedation in pediatric MRI or CT (10). There is still a lack of clinical experience in the use of DEX hydrochloride for special populations such as children, and further in-depth study is needed in terms of the safety of the medication. Although studies have reported the sedative effect of intranasal DEX vs. oral chloral hydrate for CT/MRI examinations in children, the sample size is small, and the conclusions are controversial (11, 12). Therefore, we performed a meta-analysis to systematically evaluate the efficacy and safety of intranasal DEX vs. oral chloral hydrate for CT/MR examinations in children, thus providing an evidence-based reference for clinical rational drug use.

## Methods

This meta-analysis was conducted following the Preferred Reporting Items for Systematic Reviews and Meta-Analyses (PRISMA) guideline (13).

### Literature Retrieval Strategy

Cochrane Library, PubMed, Embase, Web of Science, China National Knowledge Infrastructure (CNKI), and China WanFang were searched for relevant literature up to December 30, 2021, with language limited to English and Chinese. We aimed to collect randomized controlled trials (RCTs) investigating intranasal DEX (test group) or oral chloral hydrate (control group) for CT/MRI examinations in children. The keywords searched included: “Dexmedetomidine,” “Infant,” “Child,” “Children,” “CT,” “Computed tomography,” “MRI,” and “Magnetic Resonance Imaging.” Detailed retrieval process in PubMed was provided in Supplementary Table 1. If some important information were not provided in the original literature, we would seek it from the corresponding author through email.

### Inclusion and Exclusion Criteria

#### Inclusion Criteria

Inclusion criteria were: (1) Study design: RCTs in English or Chinese; (2) Study subjects: Pediatric patients requiring sedation for CT/MRI examinations, with the American Society of Anesthesiologists (ASA) classification of I-III; (3) Interventions: Children in the test group were given intranasal DEX, while those in the control group received oral chloral hydrate; (4) Outcome measures: the success rate of sedation, onset time of sedation, duration of sedation, time to awakening, the incidence of nausea and vomiting, the incidence of hypotension, incidence of bradycardia. Successful sedation was defined as the ability to complete all examinations after the onset of sedation, with sedation success rate = number of successful sedation/total number of cases × 100%.

#### Exclusion Criteria

Exclusion criteria were: (1) literature in which full text or data cannot be obtained; (2) duplicate publications repeatedly published literature; (3) case report, review, or animal research.

### Literature Screening and Data Extraction

Two investigators independently screened the retrieved articles for relevancy, extracted data from the studies, and cross-checked. Discrepancies were resolved by consensus with the two investigators or by consultation with a third investigator. Data extracted were included the first author, publication year, number of patients, sex, age, body mass, interventions, and outcome measures.

### Literature Quality Evaluation

Cochrane risk of bias tool version 5.1.0 was used to evaluate the quality of the included studies in terms of selection bias, selection bias, performance bias, detection bias, attrition bias, reporting bias, and other potential sources of bias. Each item was further divided into high risk, low risk, and unclear.

### Statistical Analysis

Meta-analysis was performed using Stata 15.0 software. The heterogeneity among the results of the included studies was tested using the χ^2^-test. Qualitative data were expressed as relative risk (RR) and 95% confidence interval (CI), while quantitative data were expressed as weighted mean difference (WMD) and 95% CI. If there was no statistical heterogeneity among the studies (*P* > 0.5, and *I*^2^ < 50%), the fixed-effects model (FEM) was used for analysis; otherwise, the random-effects model (REM) was selected. If seven or more studies were included, Egger’s test was performed to assess publication bias. Additionally, sensitivity analyses were performed to verify the robustness of the findings. *P* < 0.05 was considered statistically significant.

## Results

### Literature Retrieval Results and Basic Characteristics of Included Studies

**FIGURE 1:**
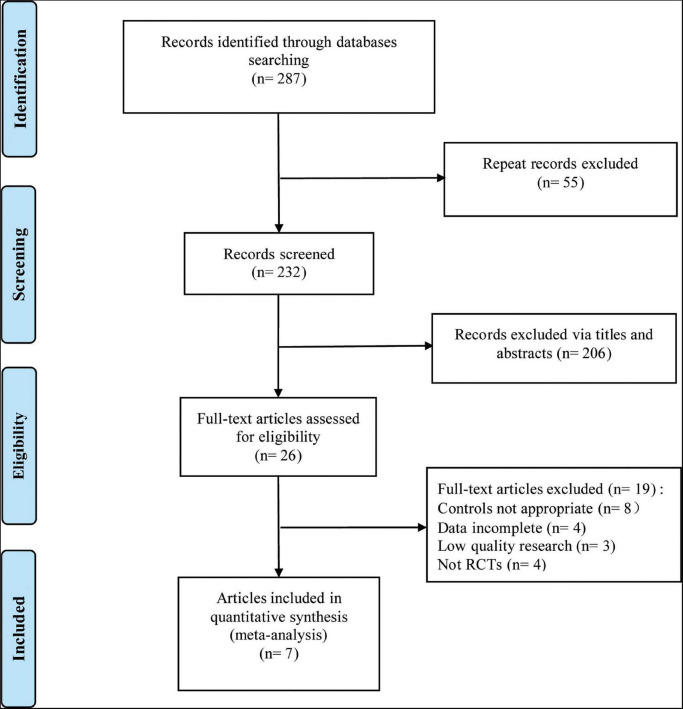
Flow diagram of literature retrieval.

**FIGURE 2:**
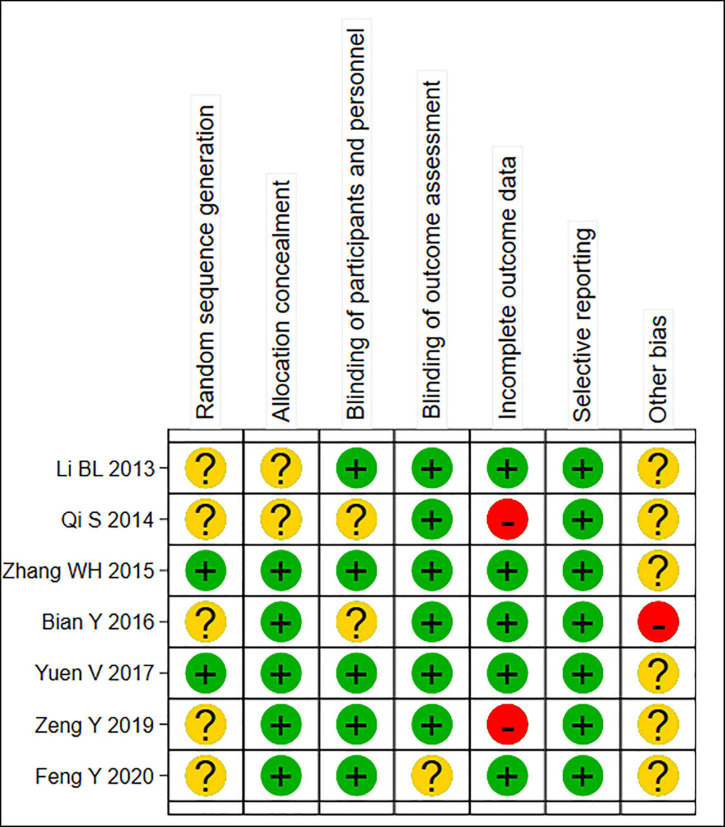
Summary risk assessment of literature bias. “Yes” indicates “low risk of bias”; “Unclear” indicates “moderate risk of bias”; “No” indicates “high risk of bias”.

A total of 7 RCTs (11, 12, 14–18) were included in this meta-analysis, with 1,846 patients (*n* = 1,325 in the test group, *n* = 521 in the control group). The literature screening process is shown in Figure 1. The basic characteristics of the included studies are shown in Table 1. The results of the quality assessment are shown in Figure 2.

**TABLE 1:** Basic characteristics of the included studies.

References	Year	Country	ASA	Average weight (kg)		Ages (month)	Interventions		Sample size (DEX/CH)	Outcomes
					
				Dex	CH		Dex (intranasal, mg/Kg)	CH (oral, mg/Kg)		
Li et al. (14)	2013	China	I,II	11.2	11	2–84	2	50	99/107	➀➁➂➃➄➆
Qi et al. (15)	2014	China	I,II,III	17.67	17.84	24–60	1.5	50–80	20/20	➀➄
Zhang et al. (11)	2015	China	I,II	3.8	3.3	1–6	1–2	25	100/50	➀➁➂➃
Bian et al. (16)	2016	China	I,II,III	6.5	7	1–12	1	25	100/100	➀➄➅
Yuen et al. (12)	2017	China	I,II	12	11.6	2–79	3	50	87/107	➄➅➆
Zeng et al. (17)	2019	China	I,II	12.15	12.95	12–36	2	60	26/26	➀➁➂➃
Feng et al. (18)	2020	China	I,II	12.25	12.87	12–36	2	60	30/30	➀➁➂➄➅

*① Success rate of sedation; ② Onset time of sedation; ③ Duration of sedation; ④ Time to awakening; ⑤ Incidence of nausea and vomiting; ⑥ Incidence of bradycardia; ⑦ Incidence of hypotension; Dex, dexmedetomidine; CH, chloral hydrate; ASA, American Society of Anesthesiologists.*

### Meta-Analysis Results

#### Success Rate of Sedation

**FIGURE 3:**
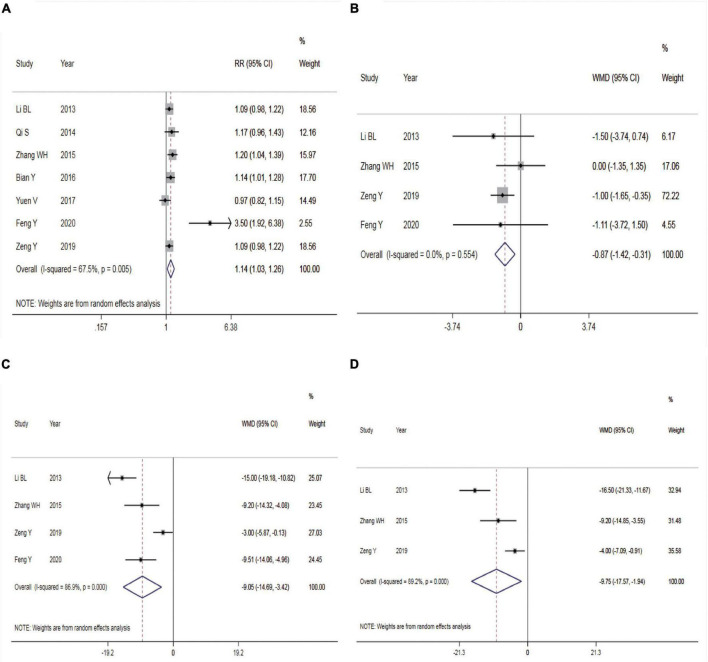
Efficacy of dexmedetomidine vs. Chloral hydrate on CT/MRI sedation in children. **(A)** Success rate of sedation; **(B)** onset time of sedation; **(C)** duration of sedation; **(D)** time to awakening. CT, Computed tomography; MRI, Magnetic Resonance Imaging.

Seven trials reported the sedation success rate. The REM was used for meta-analysis because of statistical heterogeneity among the studies (*P* = 0.005, *I*^2^ = 67.5%, Figure 3A). The result showed that intranasal DEX had a higher success rate of sedation than oral chloral hydrate (RR = 1.14, 95% CI: 1.03–1.26, *P* = 0.011). Subgroup analysis showed that the heterogeneity did not reduce in the pooled analysis of sedation success rate in ASA I-II children (Table 2). Three studies (14, 17, 18) adopted Dex dosage of 2μg/Kg with a pooled sedation success rate of 85.07%, while a pooled sedation success rate was 71.31% with chloral hydrate dosage of 50–60 mg/kg.

**TABLE 2:** Subgroup analysis.

Outcomes	ASA	*n*	RR	95%CI	*P* for RR	*I*^2^ (%)	*P* for heterogeneity	Model
Success rate of sedation	Overall	7	1.14	1.03∼1.26	**0.010**	66.9	0.006	REM
	I,II	5	1.15	1.00∼1.34	0.056	77.5	0.001	REM
	I,II,III	2	1.15	1.03∼1.27	**0.009**	0.0	0.817	FEM
Incidence of nausea and vomiting	Overall	5	0.09	0.04∼0.23	**0.000**	37.8	0.169	FEM
	I,II	3	0.04	0.01∼0.16	**0.000**	0.0	0.433	FEM
	I,II,III	2	0.38	0.09∼1.62	0.192	0.0	0.919	FEM

*ASA, American Society of Anesthesiologists; RR, relative risk; CI, confidence interval; REM, random-effects model; FEM, fixed-effects model.*

*Bold indicates that RR differences are statistically significant.*

#### Onset Time of Sedation

Four studies reported the onset time of sedation. There was no statistical heterogeneity among the studies (*P* = 0.554, *I*^2^ = 0.0%), so the FEM was used for meta-analysis (Figure 3B). The result showed a shorter onset time of sedation in children given intranasal DEX compared with those receiving oral chloral hydrate (WMD = –0.87, 95% CI: –1.42 to –0.31, *P* = 0.002).

#### Duration of Sedation

Four studies reported the duration of sedation. Statistical heterogeneity was found among the studies (*P* < 0.001, *I*^2^ = 86.9%), so the REM was employed for meta-analysis (Figure 3C). The result showed that compared with the control group, intranasal DEX achieved shorter duration of sedation (WMD = –9.05, 95% CI: –14.69 to –3.42, *P* = 0.002).

#### Time to Awakening

Three studies reported the time to awakening. The REM was adopted for meta-analysis due to statistical heterogeneity among the studies (*P* < 0.001, *I*^2^ = 89.2%, Figure 3D). The result revealed that intranasal DEX was associated with a shorter time to awakening compared with the control group (WMD = –9.75, 95% CI: –17.57 to –1.94, *P* = 0.014).

#### Incidence of Nausea and Vomiting

**FIGURE 4:**
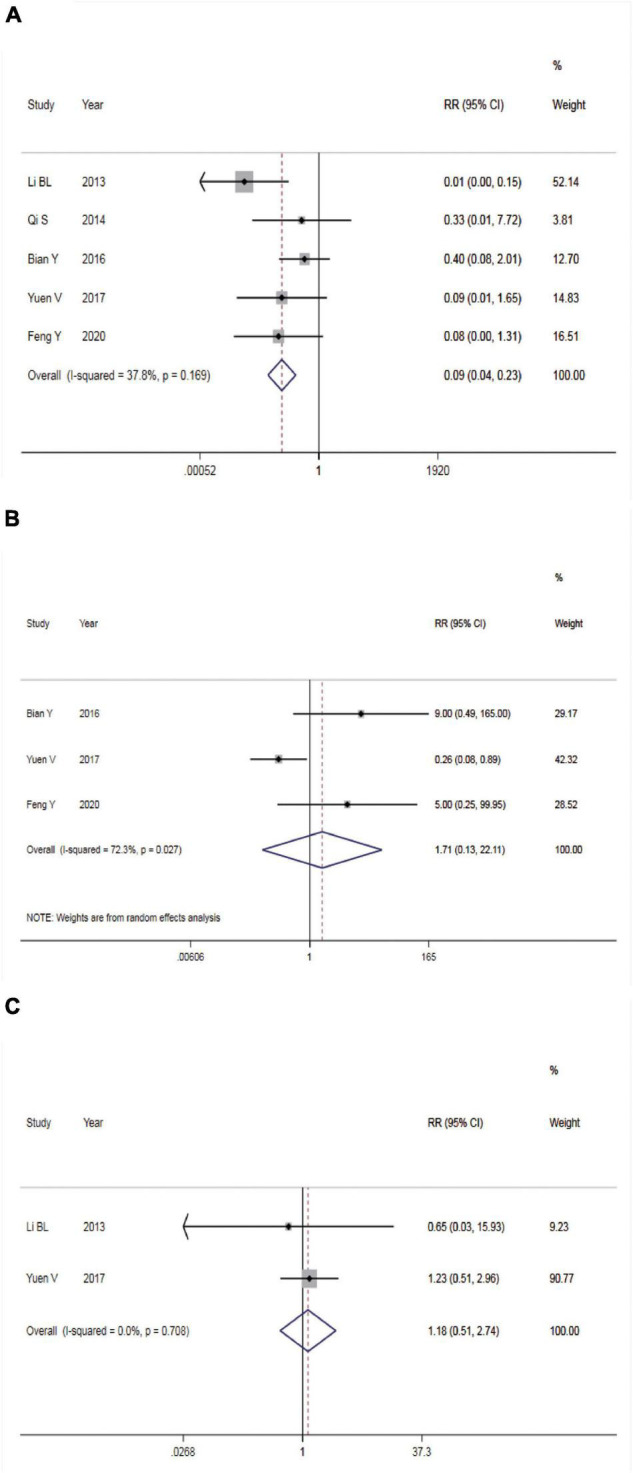
Safety of dexmedetomidine vs. Chloral hydrate on CT/MRI sedation in children. **(A)** Incidence of nausea and vomiting; **(B)** incidence of bradycardia; **(C)** incidence of hypotension.

Five studies reported the incidence of nausea and vomiting. There was no statistical heterogeneity among the studies (*P* = 0.169, *I*^2^ = 37.8%), so a FEM was utilized for meta-analysis (Figure 4A). According to the results, intranasal DEX showed a lower incidence rate of nausea and vomiting than in the control group (RR = 0.09, 95% CI: 0.04–0.23, *P* < 0.001). Subgroup analysis showed that the heterogeneity was significantly reduced in the pooled analysis of the incidence of nausea and vomiting in ASA I-II children (Table 2).

#### Incidence of Bradycardia

Three studies reported the incidence of bradycardia. The REM was used (*P* = 0.027, *I*^2^ = 72.3%) and meta-analysis result found no significant difference in the incidence rate of bradycardia between the two groups (RR = 1.17, 95% CI: 0.13–22.11, *P* > 0.05, Figure 4B).

#### Incidence of Hypotension

Two studies reported incidence of hypotension. The FEM was used (*P* = 0.708, *I*^2^ = 0.0%) and meta-analysis result found no significant difference in the incidence rate of hypotension between the two groups (RR = 1.17, 95% CI: 0.13–22.11, *P* > 0.05, Figure 4C).

### Detection of Publication Bias

Publication bias analysis was performed using the success rate of sedation as an index. As a result, the *P*-value was 0.058 for Egger’s test, suggesting no significant publication bias in this study.

### Sensitivity Analysis

**FIGURE 5:**
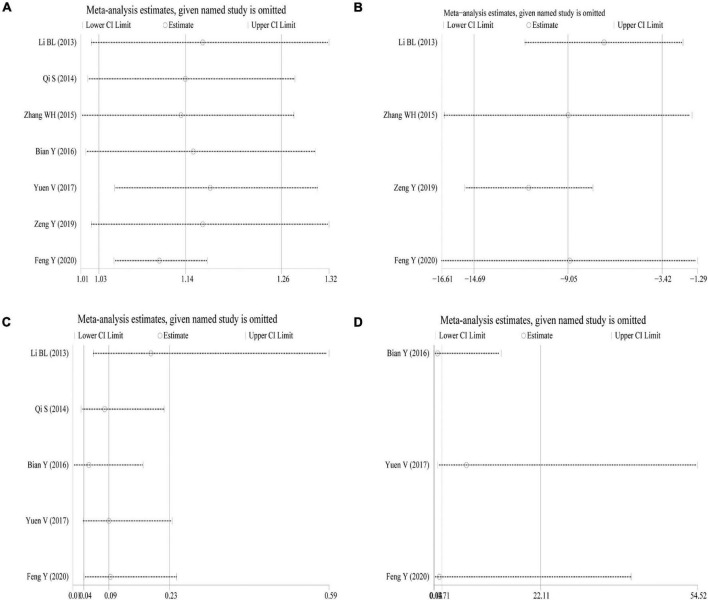
Sensitivity analysis. **(A)** Success rate of sedation; **(B)** duration of sedation; **(C)** incidence of nausea and vomiting; **(D)** incidence of bradycardia.

Sensitivity analysis was performed using the success rate of sedation, duration of sedation, the incidence of nausea and vomiting, and the incidence of bradycardia as indicators. The results showed that the effect sizes of these outcome measures did not change significantly after omitting any of the articles, indicating that the results were robust and credible (Figures 5A–D).

## Discussion

Chloral hydrate has been widely used for sedation in children and can be administered orally or rectally. It is absorbed in the gastrointestinal tract and reaches peak plasma concentrations in 30–60 min; however, this drug is not safe enough because its long-acting metabolite, trichloroethanol, has a half-life of 12–24 h and is hepatotoxic (19). Chloral hydrate can cause respiratory depression in children, accompanied by delayed sedation, nausea and vomiting (20, 21), so its application is limited. Additionally, animal experiments have found that chloral hydrate, as a γ-aminobutyric acid (GABA) receptor agonist and N-methyl-D-aspartate (NMDA) receptor antagonist, may affect brain development and induce neurotoxicity or neuronal cell apoptosis (22–24). There are increasing reports on chloral hydrate-caused adverse events of neurodevelopment in children (25). By contrast, existing animal studies have preliminarily confirmed that DEX does not act on the above receptors and may also have some protective effect against acute organ injury (26, 27). Also, some meta-analyses have shown that DEX has a protective effect on ischemic brain injury (28).

DEX acts at α2 adrenergic receptors in the locus coeruleus to induce non-rapid eye movement sleep in the natural state; this intranasal drug produces sedation with greater bioavailability and fewer adverse effects (29, 30). Intranasal administration of DEX will not make pediatric patients feel uncomfortable. In the case of sedation failure, it can also be re-administered (dose range of 1–4 μg/kg, usually 1 μg/kg). Intranasal administration of DEX has an average onset time of 30–40 min and an average time to awakening of about 90 min, and its main adverse reactions are hypotension and bradycardia, but the symptoms are mild and do not require therapeutic support (31).

This study showed that compared with the control group, intranasal DEX had a higher sedation success rate and lower onset time of sedation, duration of sedation, and time to awakening in pediatric CT/MRI examinations. It has been reported that intranasal administration of DEX can be successfully used as a rescue measure of failed chloral hydrate sedation during non-painful diagnostic procedures (32). Moreover, oral chloral hydrate can easily cause nausea, vomiting, stomach pain, respiratory depression, and other adverse reactions, which are not readily accepted by children and parents. This meta-analysis showed intranasal DEX led to a significantly lower incidence of nausea and vomiting in children. Intranasal administration of DEX is a non-invasive administration and therefore is readily accepted by children and parents. Collectively, DEX can replace chloral hydrate for sedation before pediatric examinations.

Although the subgroup analysis of ASA I-II Children showed a pooled analysis of sedation success with a *P*-value of 0.056, heterogeneity was higher than that of the overall analysis, indicating the overall pooled analysis preferable. Therefore, the results of subgroup analysis also support that, compared with oral chloral hydrate, intranasal Dex can increase the success rate of MRI/CT in children and reduce the rate of nausea and vomiting.

In terms of safety, the incidence of bradycardia and hypotension had no significant difference between the two groups. Although children showed a dose-dependent reduction in heart rate after the use of DEX, the vast majority of the decrease is within the clinically safe range and requires no medical treatment. In this study, Egger’s test on the success rate of sedation found no significant publication bias. Sensitivity analysis of the four indicators (success rate of sedation, duration of sedation, incidence of nausea and vomiting, and incidence of bradycardia) also showed that no studies that significantly changed the original conclusions and indicated robust conclusions obtained.

The dose of intranasal Dex ranged from 1 to 3 μg/kg, while the dose of chloral varied between 25 and 80 mg/kg in Children (33). A meta-analysis performed by Lewis et al. (33) exploring sedation in children with intranasal Dex showed that the sedation success rate was as high as over 85% with a Dex dosage of 2μg/kg, administration of 2μg/kg appearing to be the optimal dose. In our meta-analysis, the combined sedation success rate of three studies using 2μg/kg Dex was 85.07%, which was consistent with the findings of Lewis et al.

This study, however, still has several limitations. First, some studies did not describe the specific allocation concealment and blind method, and there may be implementation, measurement, and other biases. Second, the sample size of the included studies was small, and the power of the test may be insufficient. Third, the dose of the drugs and the age of the children are not the same, which may lead to reduced accuracy and implementability of the findings. Fourth, the included articles were all Chinese ones, causing uncertainty about the applicability of our pooled conclusions to populations in other countries. Fifth, the number of included literature of each indicator was limited, thus bringing some impact on the robustness of the conclusions. Despite the above limitations, this study is the first meta-analysis investigating the sedative effect and safety of intranasal DEX vs. oral chloral hydrate in pediatric CT/MRI examinations and has critical clinical implications.

In conclusion, intranasal DEX is superior to oral chloral hydrate for sedation and better safety during pediatric CT/MRI examinations. This meta-analysis provides evidence-based medical evidence for the clinical use of intranasal DEX for sedation before pediatric CT/MRI examinations. Considering the limitations of this study, this conclusion needs to be further validated by large-sample, multicenter, high-quality RCTs.

## Data Availability Statement

The raw data supporting the conclusions of this article will be made available by the authors, without undue reservation.

## Author Contributions

XL, YT, and XD: substantial contribution to the conception and design of the work, manuscript drafting, acquisition, analysis, and interpretation of the data, revising the manuscript critically, and final approval of the version to be published. XL and YT: critical revision of the manuscript. All authors have read and approved the final manuscript.

## Conflict of Interest

The authors declare that the research was conducted in the absence of any commercial or financial relationships that could be construed as a potential conflict of interest.

## Publisher’s Note

All claims expressed in this article are solely those of the authors and do not necessarily represent those of their affiliated organizations, or those of the publisher, the editors and the reviewers. Any product that may be evaluated in this article, or claim that may be made by its manufacturer, is not guaranteed or endorsed by the publisher.

## Abbreviations

DEX: dexmedetomidine
CT: Computed tomography
MRI: Magnetic Resonance Imaging
CNKI: China National Knowledge Infrastructure
RCTs: randomized controlled trials
WMD: weighted mean difference
RR: relative risk
PRISMA: Preferred Reporting Items for Systematic Reviews and Meta-Analyses
FEM: fixed-effects model
REM: random-effects model.
