# Supplementary material for: Efficacy and Safety of Intranasal Dexmedetomidine vs. Oral Chloral Hydrate for Sedation in Children Undergoing Computed Tomography/Magnetic Resonance Imaging: A Meta-Analysis
Source: Front Pediatr. 2022 Mar 31;10:872900. doi: 10.3389/fped.2022.872900 (PMC9008694; doi:10.3389/fped.2022.872900)
Supplement: Supplementary file 1 [file Table_1.DOCX]

**Supplementary Table 1.** Retrieval steps and results in PubMed (The retrieval time: 2021-12-30).

| Search | Query | Items found |
| --- | --- | --- |
| #1 | Dexmedetomidine [MeSH terms] | 4603 |
| #2 | "Dexmedetomidine" [Title/Abstract] | 7208 |
| #3 | #1 OR #2 | 7441 |
| #4 | Children [MeSH terms] | 1409413 |
| #5 | “Children” [Title/Abstract] OR "Child" [Title/Abstract] OR “Infant” [Title/Abstract] | 1200490 |
| #6 | #4 OR #5 | 1875557 |
| #7 | Magnetic Resonance Imaging [MeSH terms] | 494785 |
| #8 | “Magnetic Resonance Imaging” [Title/Abstract] OR “MRI” [Title/Abstract] OR “CT”[All fields] OR “Computed tomography” [All fields] | 1147891 |
| #9 | #7 OR #8 | 1324024 |
| #10 | #3 AND #6 AND #9 | 117 |
